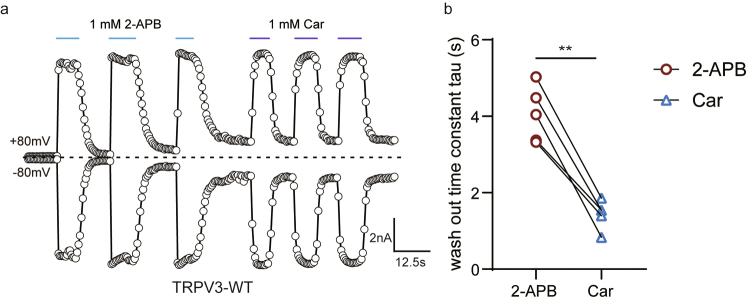# Inhibition of transient receptor potential vanilloid 3 channels by antimalarial hydroxychloroquine alleviates TRPV3-dependent dermatitis

**DOI:** 10.1016/j.jbc.2024.107733

**Published:** 2024-09-02

**Authors:** Beilei Zhang, Bo Xie, Wen Xu, Dongfan Wei, Li Zhang, Jiayi Sun, Yetan Shi, Jiangfeng Feng, Fan Yang, Heng Zhang, Xiuzu Song

**Affiliations:** 1 Department of Dermatology, Hangzhou Third People's Hospital, Zhejiang Chinese Medical University, Hangzhou, China; 2 Department of Dermatology, Hangzhou Third People's Hospital, Affiliated Hangzhou Dermatology Hospital of Zhejiang University School of Medicine, Hangzhou, China; 3 Department of Dermatology, Affiliated Hangzhou Dermatology Hospital, Zhejiang University School of Medicine, Hangzhou, China; 4 Department of Biophysics, Kidney Disease Center of the First Affiliated Hospital, Zhejiang University School of Medicine, Hangzhou, Zhejiang, China; 5 Liangzhu Laboratory, Zhejiang University Medical Center, Hangzhou, Zhejiang, China; 6 Department of Dermatology, Hangzhou Third People's Hospital, Affiliated Hangzhou Dermatology Hospital of Zhejiang University School of Medicine, Hangzhou, China

**Keywords:** TRPV3, TRP channel, electrophysiology, HCQ, dermatitis, skin inflammation

## Abstract

Transient receptor potential vanilloid 3 channel (TRPV3) is closely associated with skin inflammation, but there is a lack of effective and specific inhibitors for clinical use. In this study, we identified antimalarial hydroxychloroquine (HCQ) as a selective TRPV3 inhibitor following the prediction by network pharmacology data analysis. In whole-cell patch-clamp recordings, HCQ inhibited the current of the TRPV3 channel, with an IC50 of 51.69 ± 4.78 μM. At the single-channel level, HCQ reduced the open probability of TRPV3 and decreased single-channel conductance. Molecular docking and site-directed mutagenesis confirmed that residues in the pore domain were critical for the activity of HCQ. *In vivo*, HCQ effectively reduced carvacrol-induced epidermal thickening, erythema, and desquamation. Additionally, the serum immunoglobulin E and inflammatory factors such as tumor necrosis factor-α and interleukin-6 were markedly decreased in the dorsal skin tissues in the HCQ treatment group, as compared to the model group. Our results suggested the antimalarial HCQ may represent a potential alleviator for treating skin inflammation by inhibiting TRPV3 channels.

Transient receptor potential vanilloid 3 channel (TRPV3) is expressed in various tissues such as the brain, spinal cord neurons, and outer root sheath of hair follicles. This channel is especially abundantly expressed in the plasma membrane of keratinocytes (KCs cells) (1). Opening of TRPV3 channels mediates the nonselective influx of Ca^2+^ across the cell membrane, thereby regulating a plethora of physiological functions, including pain, itch sensation transmission, hair follicle growth regulation, and skin inflammation (2). As a polymodal sensor, TRPV3 can be activated by warm temperatures between 33 to 35 °C (3) or ligands such as natural compounds eugenol, carvacrol, thymol, camphor (4), and inorganic compound 2-aminoethoxydiphenylborate (2-APB) (5, 6). TRPV3 gain-of-function genetic variation leads to Olmsted syndrome (OS) (7) in humans and allergic dermatitis in rodents (8). OS is characterized by excessive keratinization, increased sensitivity to pain and itching sensations, and skin inflammation (9). Moreover, a recent study demonstrated that TRPV3 was overexpressed in KC cells isolated from atopic dermatitis (AD) patients' epidermis (10). Therefore, TRPV3 is a promising target for the treatment of AD.

Therefore, the discovery of TRPV3-specific inhibitors is of great clinical significance. TRPV3 inhibitors—such as forsythoside B (11), isochlorogenic acids A and B (12), citrusinine-II (13), and scutellarein (14)—have been derived mainly from active ingredients in traditional Chinese medicine. As far, none of these compounds was approved in clinical trials, which hampers the clinical investigation of TRPV3-dependent dermatitis. Thus, repurposing approved drugs for skin inflammation targeting TRPV3 is a promising alternative strategy.

Hydroxychloroquine (HCQ) is an antimalarial drug used for the treatment of a variety of autoimmune diseases such as rheumatic arthritis and systemic lupus erythematosus, juvenile idiopathic arthritis, and sicca syndrome (15). In recent years, HCQ has gradually been used in coronavirus disease 2019 (COVID-19), cancer, diabetes, heart disease, and especially skin-related diseases (16). The main mechanism of HCQ's therapeutic effect is immunomodulation, as this drug modulates the antigen presentation process of T cells (17). It also inhibits the production of interleukin (IL)-6 by T cells and monocytes (18), tumor necrosis factor-α (TNF-α), interferon-γ and other factors by monocytes (19), and calcium signaling in T cells and B cells (20).

In this study, we found HCQ was able to inhibit TRPV3 channel following the prediction of network pharmacology analysis. Single-channel recordings demonstrated that HCQ both decreased single-channel conductance and channel open probability (Po), suggesting a pore-blocker mechanism. Molecular docking–guided site-directed mutagenesis results showed residues located on the pore domain were critical for the inhibitory effect of HCQ, further supporting the pore blocker mechanism of HCQ. In addition, we further observed that *in vivo* inhibition of TRPV3 by HCQ decreases serum immunoglobulin E (IgE) level and inflammatory factors TNF-α and IL-6 in carvacrol-induced AD-like animal model. Our study suggested that the antimalarial HCQ is a potential skin inflammation alleviator by directly inhibiting TRPV3 channels.

## Result

### Network pharmacology analysis indicated TRPV3 as a potential target of HCQ

**Figure 1:**
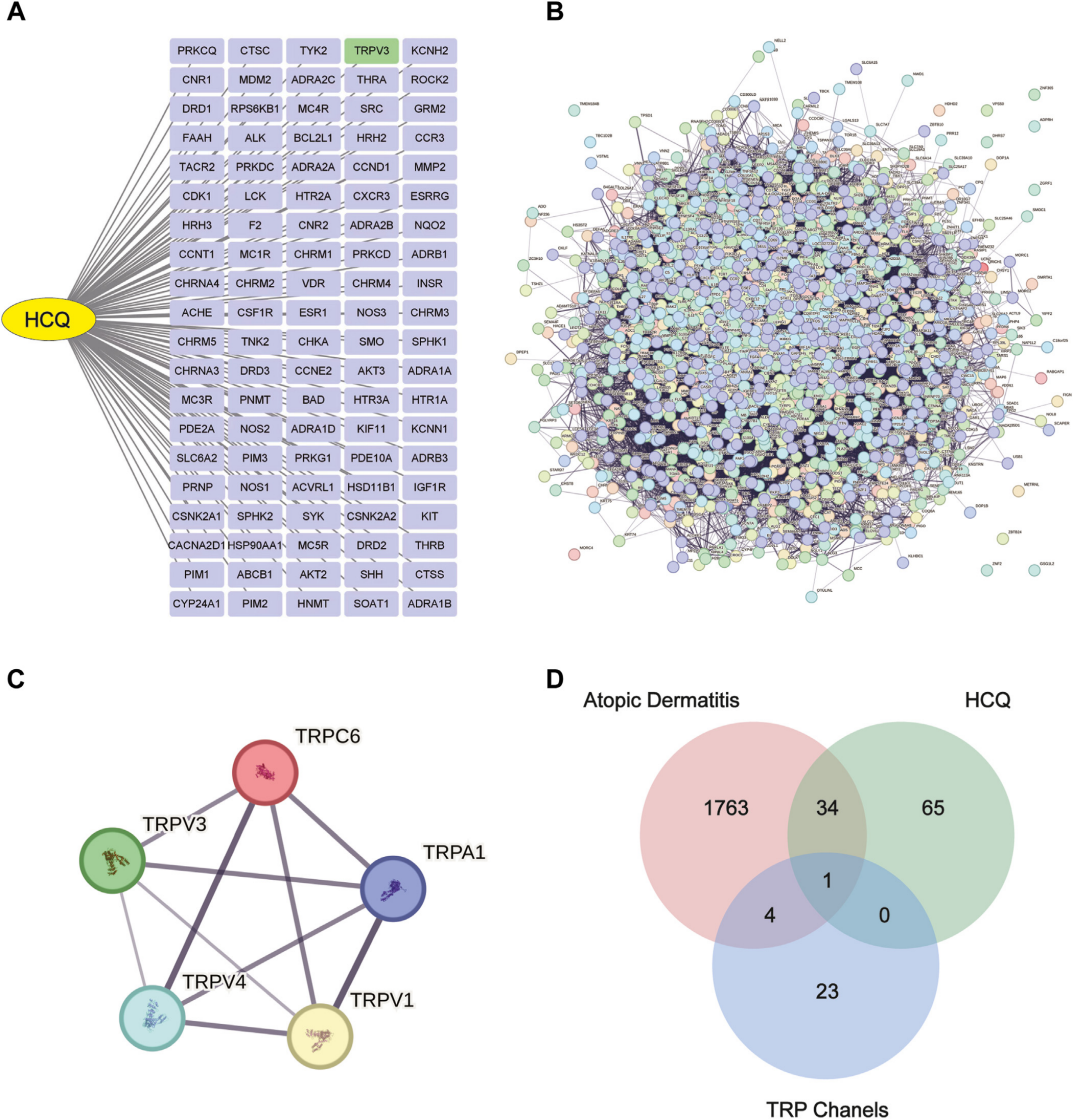
**The prediction and selection of HCQ-related targets in treating AD.***A*, the predicted targets of HCQ according to its chemical structure. *B*, the1802 AD targets and their PPI network. *C*, the extracted five TRP channels in AD-related targets and their PPI network. *D*, Venn diagram. The mutual targets among HCQ, AD and TRP channels. AD, atopic dermatitis; HCQ, hydroxychloroquine; PPI, protein–protein interaction; TRP, transient receptor potential.

We searched the SMILES of HCQ in the PubChem database. Prediction of potential targets for HCQ was performed in the Swiss Target Prediction database, which returned 100 most likely targets (Fig. 1*A*). We then retrieved AD-related targets in the GeenCards database and obtained 1802 protein nodes. We presented these proteins in the form of protein–protein interaction network through StringAPP in Cytoscape software (https://cytoscape.org) (Fig. 1*B*). Based on the pivotal role of transient receptor potential (TRP) channels in itching and skin inflammation, we extracted all TRP channels in the AD target network. A total of five TRP channels (TRPV1, TRPV3, TRPV4, TRPA1, and TRPC6) were returned (Fig. 1*C*). Finally, as we intersected the predicted targets of HCQ, AD-related targets, and TRP channels, TRPV3 was identified as the only consensus predicted protein from these three datasets (Fig. 1*D*).

### Identification of HCQ as a TRPV3 inhibitor

**Figure 2:**
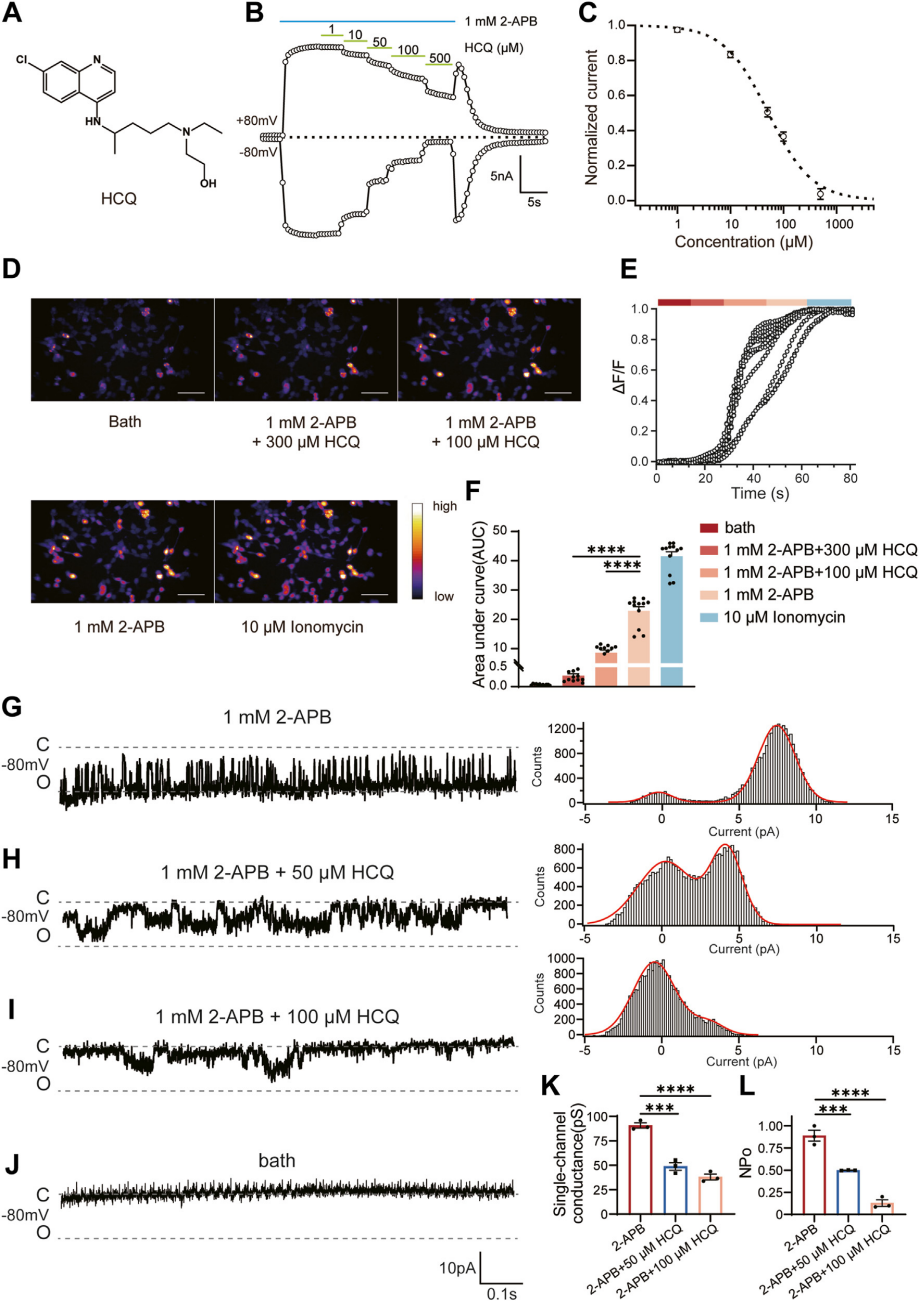
**Identification of HCQ as an inhibitor of TRPV3 channels.***A*, the chemical structure of HCQ. *B*, HCQ inhibits TRPV3 currents induced by 1 mM 2-APB in a concentration-dependent manner at ± 80 mV. *C*, the concentration–response curve of HCQ on TRPV3 channels is fitted by Hill equation. Data were shown as mean ± SEM. (n = 3 cells). *D*, calcium imaging of TRPV3-expressing HEK293T cells responded to the mixture of 1 mM 2-APB and 300 μM HCQ, the mixture of 1 mM 2-APB and 100 μM HCQ, 1 mM 2-APB or 10 μM ionomycin, respectively. The scale bar represents 50 μm. *E*, the normalized fluorescence intensity of *panel d*. *F*, summary of area under curve (AUC) from *panel E*. (mean ± SEM; n = 12 biologically independent cells), F (2, 33) = 142.80, *p* < 0.0001 compared by one-way ANOVA using Dunnett’s multiple comparisons test. ∗∗∗∗, *p* < 0.0001, compared to 2-APB. *G*, *left panel*, single-channel recordings of TRPV3 in the presence of 1 mM 2-APB at −80 mV. *Right panel*, the corresponding all-point histograms of the *left panel* are fitted to a double Gauss function (*solid line in red*), where the difference in two fitted peak values is used to calculate the single-channel conductance. *H*, *left panel*, single-channel recordings of TRPV3 in the presence of 1 mM 2-APB and 50 μM HCQ at −80 mV. *Right panel*, the corresponding all-point histograms of the left panel are fitted to a double Gauss function (*solid line in red*). *I*, *left panel*, single-channel recordings of TRPV3 in the presence of 1 mM 2-APB and 100 μM HCQ at −80 mV. *Right panel*, the corresponding all-point histograms of the *left panel* are fitted to a double Gauss function (*solid line in red*). *J*, representative single-channel recordings of TRPV3 in the bath solution at −80 mV. *K*, 50 μM HCQ and 100 μM HCQ significantly reduced the single-channel conductance of TRPV3 (n = 3 cells). Data are shown as mean ± SEM; F (2, 6) = 75.88, *p* < 0.0001 compared by one-way ANOVA using Dunnett’s multiple comparisons test. ∗∗∗, *p* = 0.0002; ∗∗∗∗, *p* < 0.0001 compared to 2-APB. *L*, 50 μM HCQ and 100 μM HCQ significantly reduced NPo of TRPV3 (n = 3 cells, mean ± SEM). F (2, 6) = 85.48, *p* < 0.0001 by one-way ANOVA using Dunnett’s multiple comparisons test. ∗∗∗, *p* = 0.0010, ∗∗∗∗; *p* < 0.0001 compared to 2-APB. 2-APB, 2-aminoethoxydiphenylborate; HCQ, hydroxychloroquine; TRP, transient receptor potential; TRPV3, transient receptor potential vanilloid 3 channel.

To confirm the inhibitory effect of HCQ on TRPV3 channels, we carried out whole-cell patch-clamp recordings. In HEK293T cells expressing TRPV3 channels, perfusion with HCQ caused concentration-dependent inhibition of TRPV3 currents induced by 1 mM 2-APB with an IC_50_ value of 51.69 ± 4.78 μM (Fig. 2, *B* and *C*). In addition, calcium imaging was also utilized to confirm the inhibition of HCQ on TRPV3 channels. Consistent with electrophysiological recordings, perfusion with HCQ effectively attenuated the intracellular calcium fluorescence induced by 1 mM 2-APB in a concentration-dependent manner (Fig. 2, *D* and *E*). These results confirmed the inhibition of HCQ on TRPV3 channels as predicted by network pharmacology analysis. We also quantified the amount of calcium influx by calculating the area under the curve (AUC) (Fig. 2*F*). Compared with the AUC of 2-APB activation (22.92 ± 1.44), 100 μM HCQ significantly inhibited calcium ion entry with an AUC of 8.65 ± 0.83 and 300 μM HCQ virtually eliminated all calcium entry with an AUC of 0.28 ± 0.05, while the AUC of positive control ionomycin was 41.59 ± 1.50.

To investigate the inhibitory mechanism of HCQ, we performed single-channel recordings of TRPV3 in the inside-out configuration (Fig. 2, *G*–*J*). In the presence of 1 mM 2-APB, TRPV3 channel Po reached 0.89 ± 0.06, 50 μM HCQ significantly decreased Po to 0.50 ± 0.03, and 100 μM HCQ significantly decreased Po to 0.13 ± 0.04 (Fig. 2*L*). On the other hand, the single-channel conductance was also decreased by HCQ from 90.69 ± 2.67 pS to 37.97 ± 2.96 pS (Fig. 2*K*). These results suggested that HCQ may inhibit channel activation through a pore-blocking mechanism. Taken together, our results demonstrated that HCQ was a novel TRPV3 inhibitor.

### Selective inhibition of TRPV3 by HCQ

**Figure 3:**
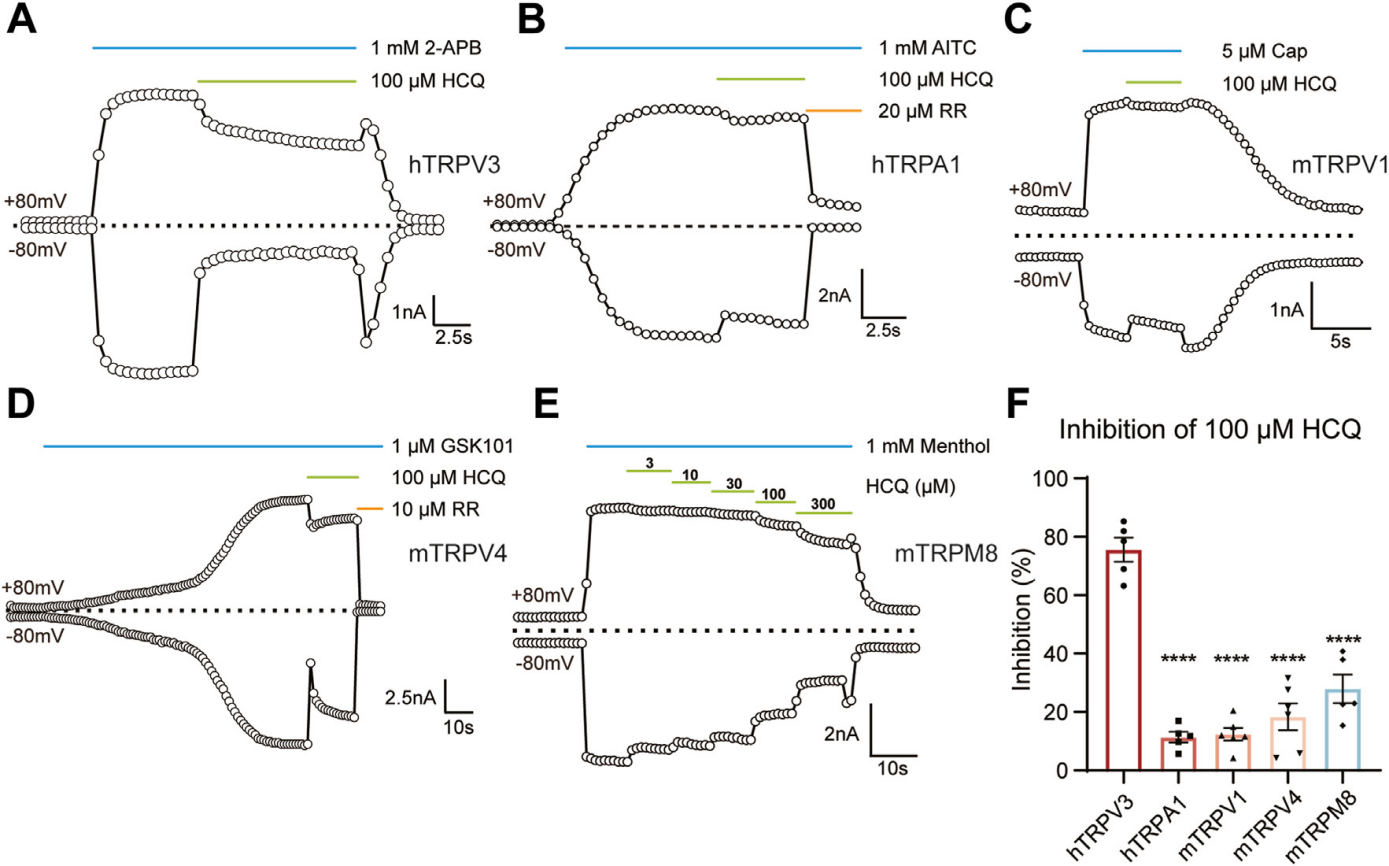
**Effect of HCQ on skin-expressed TRP channels.***A*–*E*, representative currents of hTRPV3, hTRPA1, mTRPV1, mTRPV4, and mTRPM8 at ± 80 mV activated by 2-APB (1 mM), allyl isothiocyanate (1 mM), capsaicin (5 μM), 2-APB (1 mM), GSK1016790A (1 μM), and menthol (1 mM), respectively. HCQ (100 μM) was applied after the currents reached a steady state as indicated. *F*, quantification of the inhibitory ratio of HCQ (100 μM) on hTRPV3, hTRPA1, mTRPV1, mTRPV4, and mTRPM8. Data were represented as mean ± SEM. (n = 5–6 cells). F (4, 22) = 49.00, *p* < 0.0001 by one-way ANOVA using Dunnett’s multiple comparisons test. ∗∗∗∗, *p* < 0.0001 compared to hTRPV3. 2-APB, 2-aminoethoxydiphenylborate; HCQ, hydroxychloroquine; TRP, transient receptor potential; TRPV3, transient receptor potential vanilloid 3 channel.

To elucidate the specificity of HCQ inhibition on TRPV3, we conducted whole-cell current recordings on other known pruritus-related TRP channels, such as hTRPA1, mTRPV1, mTRPV4, and mTRPM8 (Fig. 3, *A* and *F*). We observed that 100 μM HCQ reduced the current of hTRPA1 activated by 500 μM allyl isothiocyanate only by 11.37% ± 4.10% (Fig. 3*B*), and the current of mTRPV1 activated by 5 μM capsaicin by 12.37% ± 5.32% (Fig. 3*C*), mTRPV4 current activated by 1 μM GSK1016790A by 18.37% ± 11.21% (Fig. 3*D*), and mTRPM8 current activated by 1 mM menthol by 27.94% ± 10.91% (Fig. 3*E*). Because of the relatively larger inhibition of HCQ on mTRPM8 current, we tested the concentration dependence of HCQ on mTRPM8 channels. The measured IC_50_ was 262.73 ± 88.6 μM, which was more than 5-fold larger than the IC_50_ of hTRPV3. Therefore, we concluded that the inhibitory effect of HCQ on TRPV3 was superior to other skin-expressed TRP channels.

### Residues located at the pore domain are critical for the inhibition of HCQ

**Figure 4:**
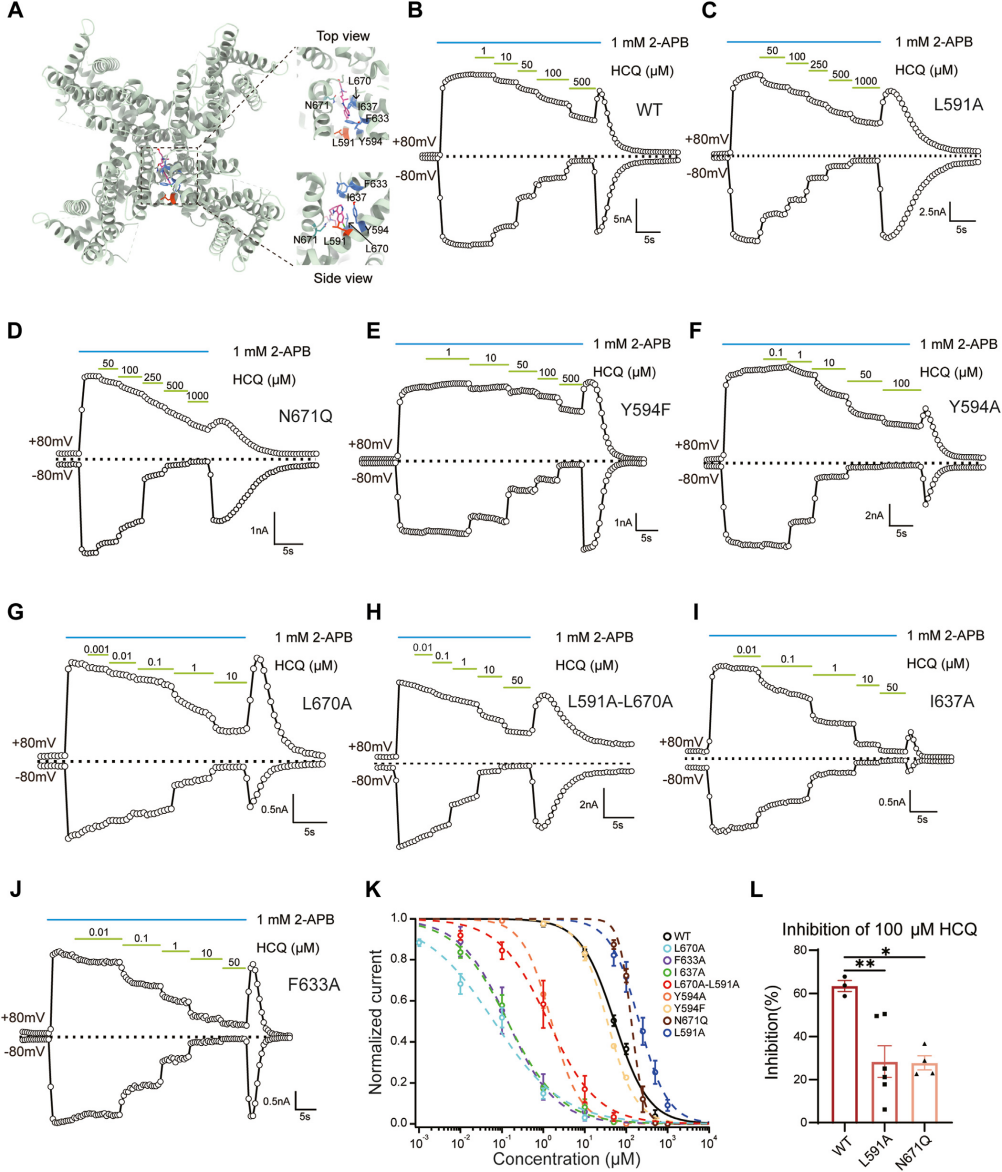
**Residues of the pore domain are critical for HCQ inhibition on TRPV3.***A*, *left panel*, docking model of HCQ to the pore domain of TRPV3 structure (6uw4). *Right panels*, *top view and side view* of the HCQ-binding pocket. *B*-*J*, representative whole-cell recording of TRPV3 and mutants in response to 1 mM 2-APB, the mixture of 1 mM 2-APB and concentration gradients of HCQ with the holding potential of ± 80 mV. *K*, concentration–response curves of HCQ in WT TRPV3 and mutants (n = 3–5 cells). *L*, quantification of the inhibitory ratio of HCQ (100 μM) on WT, L591A, and N671Q. Data are presented as mean ± SEM (n = 3–6 cells); F (2, 10) = 8.115, *p* = 0.0081 by one-way ANOVA using Dunnett’s multiple comparisons test. ∗∗, *p* = 0.0070 WT *versus* L591A; ∗, *p* = 0.0100 WT *versus* N671Q. 2-APB, 2-aminoethoxydiphenylborate; HCQ, hydroxychloroquine; TRP, transient receptor potential; TRPV3, transient receptor potential vanilloid 3 channel.

As we mentioned above, HCQ may act as a pore blocker of TRPV3. To probe the binding configuration of HCQ to TRPV3, we carried out molecular docking using Rosetta suite (https://rosettacommons.org) (21) and validated the docking models with point mutations in TRPV3 (Fig. 4*A*). Site-mutagenesis showed L591 and Y594 at S5, F633, and I637 at pore helix, L670 and N671 located at S6 were critical in the inhibition by HCQ. Especially, L591A led to a significant right shift on the concentration–response curve of HCQ with the IC_50_ of 203.06 ± 10.80 μM (Fig. 4*C*). And N671Q changed the concentration–response curve of HCQ with the IC_50_ to 132.59 ± 8.6 μM (Fig. 4*C*). 100 μM HCQ merely showed 28.36% ± 7.31% inhibition on L591A mutant and 27.77% ± 3.30% inhibition on N671Q mutant as compared to 75.60% ± 4.13% on WT TRPV3 channels (Fig. 4*L*). On the other hand, alanine mutations on Y594, F633, I637, and L670 caused dramatically left shift on the concentration–response curves of HCQ with the IC50 of 1.57 ± 0.08 μM, 0.14 ± 0.01 μM, 0.14 ± 0.02 μM, and 0.17 ± 0.04 μM, respectively (Fig. 4, *F*, *G*, *I*, and *J*). Notably, Y594F mutation showed similar concentration dependence to WT TRPV3 in response to HCQ with the IC_50_ of 34.83 ± 1.59 μM (Fig. 4*E*), suggesting the benzene ring in the side chain of Y594 was critical for the activity of HCQ. Furthermore, when we introduced L670A to L591A mutant, we also observed a left shift on the concentration–response curve on this double mutant as compared to L591A mutant (Fig. 4*H*). These results demonstrated HCQ binds to the pore domain to inhibit TRPV3 channels, further supporting the pore-blocking mechanism of HCQ.

### HCQ can alleviate carvacrol-induced dermatitis

**Figure 5:**
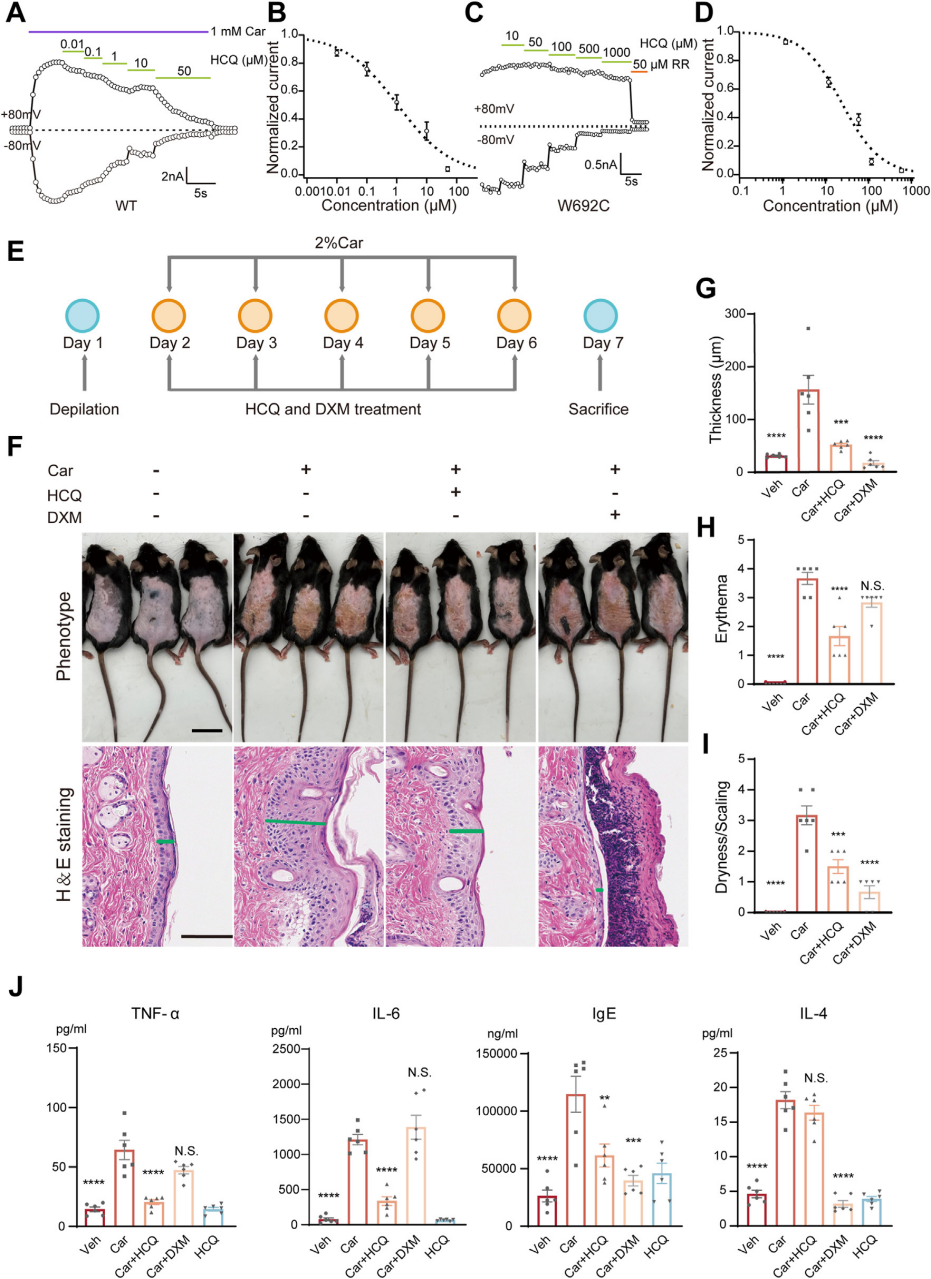
**HCQ can attenuate mice back skin lesions induced by TRPV3 agonist carvacrol.***A*, representative whole-cell recording of TRPV3 in response to 1 mM carvacrol (Car), the mixture of 1 mM Car, and concentration gradients of HCQ with the holding potential of ± 80 mV. *B*, concentration–response curve of Car and HCQ on TRPV3 channels fitted by Hill equation. Data were shown as mean ± SEM (n = 6 cells). *C*, representative whole-cell recording of W692C mutants in response to concentration gradients of HCQ with the holding potential of ± 80 mV. *D*, concentration–response curve of HCQ on W692C mutants fitted by Hill equation. Data were shown as mean ± SEM (n = 4 cells). *E*, paradigm of generation of AD-like mice model induced by Car. *F*, representative images of the gross appearance (*upper panel*) and H&E-stained sections (*bottom panel*) of the dorsal skins of WT C57BL/6J mice treated with vehicle (Veh, 30% alcohol as solvent), 2% Car, a mixture of Car and HCQ (2 mg/ml), and a mixture of Car and DXM (1 mg/ml). A 200 μl volume of drug was applied to each mouse. *Green lines* indicate the thickness of the epidermis. *Upper panel*, the scale bar represents 2 cm; *bottom panel*, scale bar represents 100 μm. *G*–*I*, quantification of thickness (G), erythema (H), dryness, and scaling (I), (n = 6 mice, mean ± SEM), thickness: F (3, 20) = 20.68, *p* < 0.0001; erythema: F (3, 20) = 55.10, *p* < 0.0001; dryness and scaling: F (3, 20) = 39.61, *p* < 0.0001 by one-way ANOVA followed using Tukey's post hoc test. N.S., no significance; ∗∗∗, *p* < 0.001; and ∗∗∗∗, *p* < 0.0001 compared to Car. *J*, relative mRNA levels of TNF-α, IL-6, IgE, and IL-4 in the mice dorsal skins of the indicated groups. Data were shown as mean ± SEM (n = 6 mice), TNF-α: F (3, 20) = 26.08, *p* < 0.0001; IL-6: F (3, 20) = 42.45, *p* < 0.0001; IgE: F (3, 20) = 15.66, *p* < 0.0001; IL-4: F (3, 20) = 76.11, *p* < 0.0001 by one-way ANOVA followed by Tukey's post hoc test. N.S., no significance; ∗∗, *p* = 0.0053; ∗∗∗, *p* < 0.001; and ∗∗∗∗, *p* < 0.0001 compared to Car. AD, atopic dermatitis; DXM, dexamethasone; HCQ, hydroxychloroquine; IgE, immunoglobulin E; IL-6, interleukin-6; TNF-α, tumor necrosis factor-α; TRP, transient receptor potential; TRPV3, transient receptor potential vanilloid 3 channel.

A recent study suggested a specific TRPV3 activator carvacrol was able to cause skin inflammation, which was attenuated in TRPV3 ^−/−^ mice (14). To test the effect of HCQ on carvacrol-induced skin inflammation, we evaluated the inhibitory effect of HCQ on TRPV3 activated by carvacrol *in vitro*. As shown in Figure 5, *A* and *B*, HCQ also inhibited TRPV3 current induced by carvacrol in a concentration-dependent manner with an IC50 of 1.07 ± 0.38 μM. In addition, HCQ also inhibited the activity of a gain-of-function TRPV3 mutant W692C (Fig. 5, *C* and *D*), which caused OS for its spontaneously high-Po and thus led to spontaneous dermatitis (22). These results indicated that HCQ may attenuate TRPV3-mediated skin inflammation by inhibiting TRPV3 channel activity.

We therefore generated a carvacrol-induced AD mouse model to test the effect of HCQ. To generate carvacrol-induced AD mouse model (Figs. 5*E* and 1 day after depilation using hair removal cream, 2% carvacrol (200 μl, dissolved in 30% ethanol solution) or in the presence of 2 mg/ml HCQ and 1 mg/ml dexamethasone (DXM was topically smeared onto mouse dorsum (once a day, for five consecutive days). Treatment of mice with 2 mg/ml of HCQ significantly alleviated dermatitis manifestations on the dorsal skin (Fig. 5*F*, upper panel).

We conducted H&E staining of the mouse epidermis (Fig. 5*F*, bottom panel), revealing pronounced thickening of the stratum corneum in the model group, accompanied by loosening of the spinous layer and congestion and edema of KC cells. Based on H&E staining results, we measured epidermal thickness and quantified it (Fig. 5*G*). We found that the epidermis in the model group thickened to 156.10 ± 27.18 μm, significantly thicker than that in the HCQ-treated group (51.49 ± 3.01 μm), while the DXM-positive control group exhibited thinning of the stratum corneum to 16.81 ± 4.54 μm, possibly due to hormonal effects. Carvacrol induced an increase in dry desquamation score and erythema scores in mice to 3.17 ± 0.31 and 3.67 ± 0.21, respectively, whereas the HCQ treatment group exhibited a significant decrease in dry desquamation scores to 1.50 ± 0.22 (Fig. 5*I*) and erythema scores to 1.67 ± 0.33 (Fig. 5*H*). However, the erythema score in the DXM group was 2.83 ± 0.17, showing no significant decrease, possibly due to excessive thinning of the stratum corneum and visibility of subcutaneous vessel (23).

In recent years, it has been found that TRPV3 channels on KC cells are involved in skin inflammation by activating the NF-κB signaling pathway, and mainly release Th1-type inflammatory factors such as IL-6, IL-8, and TNF-α (24). Furthermore, we measured inflammatory factors in mouse tissues and found that HCQ significantly inhibited the release of carvacrol-induced TNF-α and IL-6 by 88.10% ± 4.12% and 77.09% ± 5.38% (Fig. 5*J*), respectively. Serum IgE levels were also well suppressed by 60.22 ± 11.26% (Fig. 5*J*). The IL-4 levels in HCQ-treated mouse tissues showed no significant difference compared to the model group, whereas the DXM-positive control group effectively reduced IL-4 levels (Fig. 5*J*). These data indicate that HCQ can alleviate carvacrol-induced skin inflammation and epidermal thickening in mice.

## Discussion

**Figure 6:**
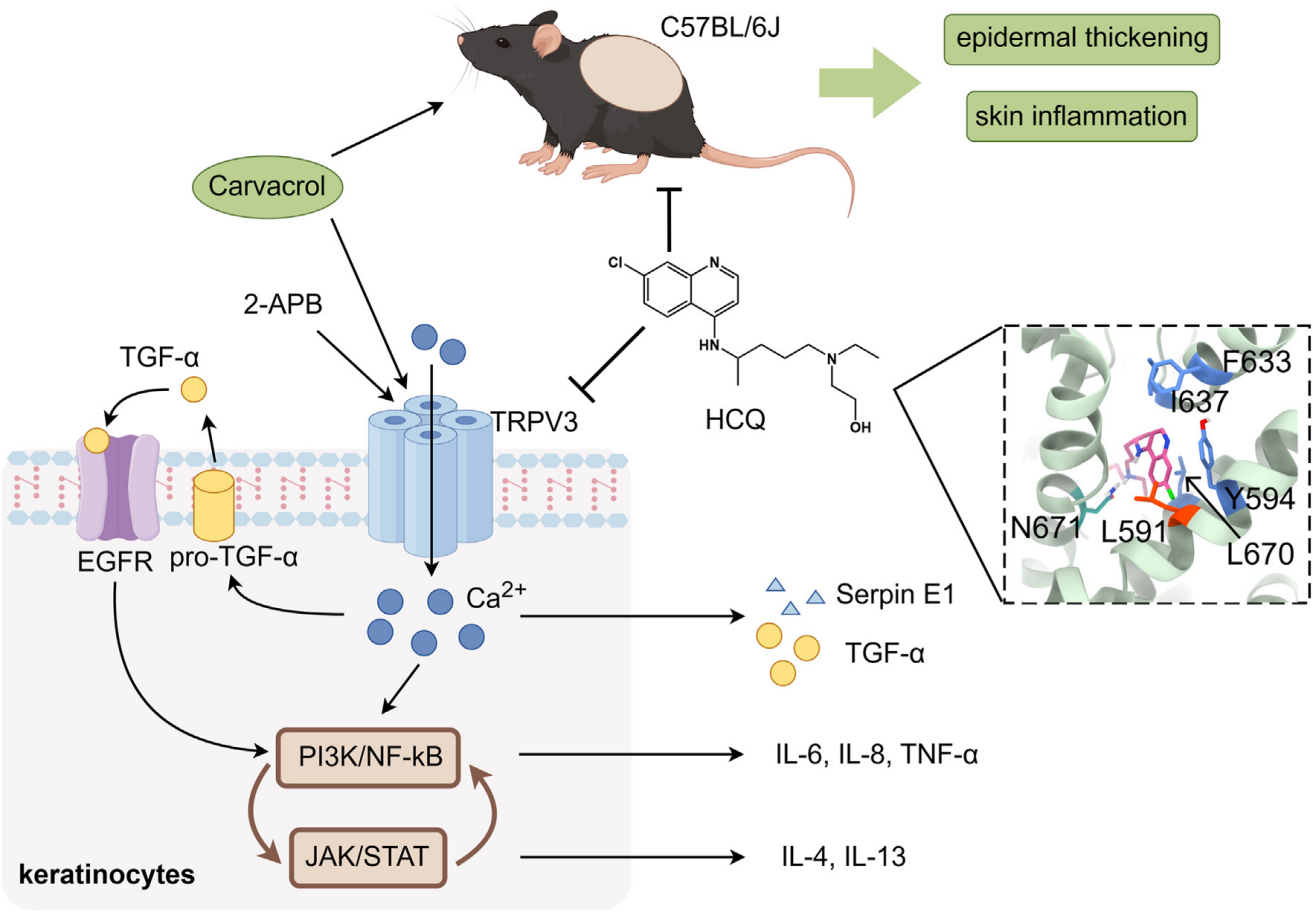
**Schematic diagram of inhibition of TRPV3 by HCQ alleviating skin inflammation in mice.** Carvacrol activates TRPV3 to cause Ca^2+^ influx into keratinocytes, leading to the release of TGF-α and its subsequent binding to EGFR, the activation of PI3K/NF-κB pathway, and release of inflammatory factors such as IL-6, IL-8, and TNF-α. In contrast, HCQ inhibits the opening of TRPV3 by binding to the pocket around N671 and L591 and opposes the effects of carvacrol, which attenuates the thickening of mouse dorsal skin and reduces the release of inflammatory factors in the tissues. AD, atopic dermatitis; EGFR, epidermal growth factor receptor; HCQ, hydroxychloroquine; IL, interleukin; TNF-α, tumor necrosis factor-α; TRP, transient receptor potential; TRPV3, transient receptor potential vanilloid 3 channel.

In this study, we identified HCQ as the selective inhibitor of TRPV3, mitigating skin inflammation and epidermal thickening caused by TRPV3 activation. Molecular docking and site-directed mutagenesis further elucidated the key residues involved in the interaction between HCQ and TRPV3. Our findings uncovered a new target for the anti-inflammatory mechanism of HCQ and provided new insights into its therapeutic applications, suggesting its potential in the development of novel drugs for skin inflammation (Fig. 6).

Our single-channel recordings demonstrated that HCQ inhibited TRPV3 by reducing channel Po and decreasing single-channel conductance. Additionally, we identified mutations at the pore domain that significantly weaken the inhibitory effect of HCQ. Combined with docking results, HCQ likely binds to S5 and S6, with its tail extending into the central pore region, thereby inhibiting TRPV3 through a pore-blocking mechanism. TRPV3 can be suppressed by Mg^2+^, exhibiting a decrease in single-channel conductance, whereas the Po of TRPV3 remains unchanged (25), suggesting the pore-blocking mechanism of HCQ may be partially similar to Mg^2+^. Intriguingly, we also observed an off-response upon washout in both WT and mutants, akin to the phenomenon seen during H^+^ activation of TRPV3 and TRPV1 (26). H^+^ exerts dual effects on TRPV1 and TRPV3. Po of TRPV1 and TRPV3 at low pH could reach the maximum level, but inhibition in single-channel conductance contributed to the decline of overall current amplitude. Washout of proton removed conductance inhibition and led to a transient recovery of activation current. Similarly, the presence of the off-response for HCQ inhibition was due to the much slower gating transition than the wash-off speed of HCQ (27). Intriguingly, the off-response was indiscernible when TRPV3 was activated by carvacrol. To explain that, we measured the wash-off speed of 2-APB or carvacrol. We observed that the wash-off speed was much faster for carvacrol than 2-APB (Figs. S3*A* and S3*B*), and the wash-off time constant was calculated to be 1.43 ± 0.38 s for carvacrol and 4.05 ± 0.73 s for 2-APB, respectively (Fig. S3*B*). The faster wash-off of carvacrol may lead to a faster channel gating transition, where the off-response may be more difficult to observe.

We confirmed the inhibitory effect of HCQ on 2-APB and carvacrol activation of TRPV3, with HCQ demonstrating a lower IC50 against carvacrol. Since 2-APB exhibits nonspecific excitatory effects on TRPV2, TRPV3, and TRPV1, while carvacrol displays good selectivity on TRPV3 and induces characteristic AD-like dermatitis in mice, making it a proper tool compound to test the effect of HCQ *in vivo*. In this study, HCQ significantly reduced the elevation of Th1-type inflammatory factors IL-6 and TNF-α induced by carvacrol. IL-6 and TNF-α play a crucial role in AD (28). Activation of the NF-κB inflammatory pathway in AD further releases proinflammatory cytokines such as IL-1β, IL-6, and IL-8, contributing to the regulation of the IL-4/signal transducer and activator of transcription 6 inflammatory pathway (29). Therefore, it is possible that HCQ inhibits calcium influx through TRPV3, which suppresses downstream signaling of KC cells, inhibits the NF-κB inflammatory pathway, and reduces the release of inflammatory factors such as IL-6 and TNF-α, thereby attenuating the cascade amplification of inflammation. In addition, IgE is recruited by lymphocytes to release mast cells and activate B cells (30), and serum IgE levels often reflect the severity of AD (31). HCQ effectively inhibits serum IgE levels. As a control group, DXM also reduces serum IgE levels, primarily by reducing Th2-type inflammatory factor IL-4 (32), while showing no significant effect on IL-6 and TNF-α. Furthermore, activation of TRPV3 leads to the release of TGF-α, which activates epidermal growth factor receptor, phosphorylating the PI3K/NF-κB pathway, and results in the proliferation of epidermal KC cells (33, 34). Consistent with this, our statistical analysis of mouse appearance and H&E staining showed that HCQ alleviates the increase in the thickness of the mouse dorsal stratum corneum induced by carvacrol and reduces erythema and scaling scores.

In conclusion, we demonstrated that HCQ could inhibit TRPV3 opening in the form of pore blocking, thereby relieving the symptoms of dermatitis and reducing the release of inflammatory cytokines in mice, suggesting the potential of HCQ in the treatment of AD.

## Experimental procedures

### Target prediction and screen of HCQ

The SMILES chemical structure of HCQ was downloaded from the PubChem website (https://pubchem.ncbi.nlm.nih.gov/). The Swiss Target Prediction database (http://www.swisstargetprediction.ch/) was used to predict the potential targets of HCQ. The top 100 targets were returned from the database according to the SMILES structure. Targets related to AD were retrieved from the database of Genecards (https://www.genecards.org/) by searching with the keyword of “AD” and with the species of “*Homo sapiens*.” Protein–protein interaction networks were analyzed on the Cytoscape software (version 3.10.1) with StringAPP. The intersection of datasets as well as the Venn diagram was performed with jvenn (35) (https://jvenn.toulouse.inra.fr/app/example.html).

### Animals

To investigate the effect of HCQ on carvacrol-induced dermatitis in animals, C57BL/6J male mice (aged 6 weeks) were obtained from the Animal Experimental Center of Hangzhou Medical College (Hangzhou, Zhejiang Province). Mice were housed and maintained in an environment with a 12-h light/dark cycle and a controlled temperature of 22.0 ± 2.0 °C. Food and water were accessible *ad libitum*. All the animal procedures were approved by the Institutional Animal Care & Use Committee of Zhejiang Center of Laboratory Animals (ZJCLA).

### Cell culture and transfection

HEK293T cells are cultured using Dulbecco's modified Eagle's medium containing 10% fetal bovine serum and 1% penicillin-streptomycin in a cell incubator with 5% CO_2_ at 37  °C. Plasmids of murine TRPV1, TRPV4, TRPM8, and human TRPV3, TRPA1 are labeled with YFP or GFP as indicators for subsequent electrophysiological recordings. Complementary DNA constructs of ion channels were transiently transfected with Lipofectamine 3000 (Invitrogen). And 12 to 18 h after transient transfection, electrophysiological recordings were performed.

### Generation of AD-like animal model by carvacrol

To generate an AD-like mice model, 2% carvacrol (dissolved in 30% ethanol solution) was topically smeared onto the mice dorsum 1 day after hair removal with a depilatory cream (Vetin Depilatory Cream). Mice received a topical administration of an equal volume (200 μl) of 2% carvacrol or in the presence of 2 mg/ml HCQ. Mice that underwent topical application of 2% carvacrol containing 1 mg/ml DXM or 30% ethanol solution were used as positive control and vehicle group, respectively. After the application of carvacrol for 5 days, the external appearance of mice was photographed and then sampled. The external appearance was graded based on three aspects: thickness, erythema/bleeding, and dryness/desquamation, with severity assessed on a score of 0 to 4. The degree of thickness was measured by the thickness of the skin in H&E staining. Skin tissues were either fixed in 4% paraformaldehyde or placed in PBS buffer containing 1% protease inhibitor, facilitating subsequent steps of paraffin embedding or ELISA experiments.

### Histological sections of skin tissues

Mice were sacrificed 24 h after the last dose of treatment. Mouse dorsal skin was removed with scissors and forceps before being fixed with 4% paraformaldehyde. Paraffin-embedded tissues were sectioned and stained with H&E. Pictures of stained sections were acquired with a Nikon Eclipse Ti-S microscope with a charge-coupled device camera (DS-Ri2, Nikon).

### ELISA assay

Animal skin tissues were placed in PBS buffer containing 1% protease inhibitors, then homogenized, and centrifuged for supernatants. Cytokine concentrations of supernatants (IL-4, IL-6, TNF-α, and IgE) were measured using ELISA kits (Tsingke) according to the manufacturer's instructions. The absorption at 450 nm was measured by a microplate reader (Thermo Multiskan MK3).

### Electrophysiology

Patch-clamp recordings were carried out with a HEKA EPC10 amplifier controlled by PatchMaster software (HEKA; https://www.heka.com/products/products_main.html#soft_pm) (36). Patch pipettes were prepared from borosilicate glass and fire-polished to the resistance of 3 to 8 MΩ by P-97 puller for whole-cell recordings. For single-channel recordings, patch pipettes were fire-polished to a higher resistance of 6 to 10 MΩ. Both bath solution and pipette solution contained 130 mM NaCl, 10 mM glucose, 0.2 mM EDTA, and 3 mM Hepes and were adjusted to pH 7.2 to 7.4 with NaOH. Whole-cell recordings and single-channel recordings were performed at ± 80 mV. The current was sampled at 10 kHz and filtered at 2.9 kHz. All recordings were performed at room temperature (25  °C). A gravity-driven system (RSC-200, Bio-Logic) with freely rotated perfusion tubes was used for the perfusion of ligands. Bath and ligand solutions were delivered through separate tubes to minimize the mixing of solutions. Patch pipette holding cells were placed in front of the perfusion tube outlet for perfusion. Igor Pro versions 5.05 and 9.05 (WaveMetrics; https://www.wavemetrics.com/products/igorpro) were used to analyze the data.

### Calcium imaging

Calcium imaging assays were performed as described previously (37). Transiently transfected HEK293 cells seeded on 25 mm coverslips were washed twice with an extracellular solution containing 140 mM NaCl, 5 mM KCl, 2 mM MgCl_2_, 2 mM CaCl_2_, 10 mM glucose, and 10 mM Hepes (pH 7.4), followed by incubation in 2 ml of extracellular solution supplemented with 2 μM Fluo-4/AM as a Molecular Probes at room temperature for 60 min. Fluorescence images of HEK293T cells were acquired with a Nikon Eclipse Ti2 microscope with an optiMOS charge-coupled device camera controlled by the Ocular Software (Molecular Devices; https://www.photometrics.com/products/ocular). Fluo-4 AM was excited at 500/20-nm excitation, and fluorescence emission was detected at 535/30-nm. Fluorescence images were analyzed with Fiji software (https://fiji.sc/), GraphPad Prism (https://www.graphpad.com/scientific-software/prism/www.graphpad.com/scientific-software/prism/), and Office Excel (https://www.microsoft.com/en-us/microsoft-365/excel?ocid).

### Molecular docking

Molecular docking was performed as previously described (38). In brief, the Rosetta Ligand procedure of Rosetta program suite version 2019 was used to perform molecular docking of HCQ to TRPV3, and Monte Carlo algorithm was employed for sampling. The 3D conformation of HCQ was generated by Frog2 server (39). The structure of TRPV3 (PDB ID: 6UW4) was relaxed by the Relax application in Rosetta program suite. HCQ was put in the pore domain of TRPV3, and the top ten models with the lowest binding energy from 30,000 generated models were chosen for further analysis. Rosetta’s Residue_energy_breakdown application was used to extract binding energies between HCQ and TRPV3.

### Data analysis

All data from whole-cell recordings were analyzed in Igor Pro (WaveMetrics). IC50 values were determined by fitting the Hill equation to concentration-response relationships. Data are shown as mean ± SEM. Statistical significance between two groups was conducted with an unpaired two-tailed *t* test, and statistical significance among multiple groups was conducted with one-way ANOVA using Dunnett’s multiple comparisons test or Tukey's post hoc test. Differences were regarded as statistically significant with ∗, *p* < 0.05; ∗∗, *p* < 0.01; ∗∗∗, *p* < 0.001; ∗∗∗∗, and *p* < 0.0001; N.S., no significance.

## Data availability

All data needed to evaluate the conclusions in the paper are present in the paper. Additional data is available from authors upon request.

## Supporting information

This article contains supporting information (40, 41, 42, 43, 44, 45, 46, 47, 48, 49, 50).

## Conflict of interest

The authors declare that they have no conflicts of interest with the contents of this article.

## Figures and Tables

**Fig. S1:**
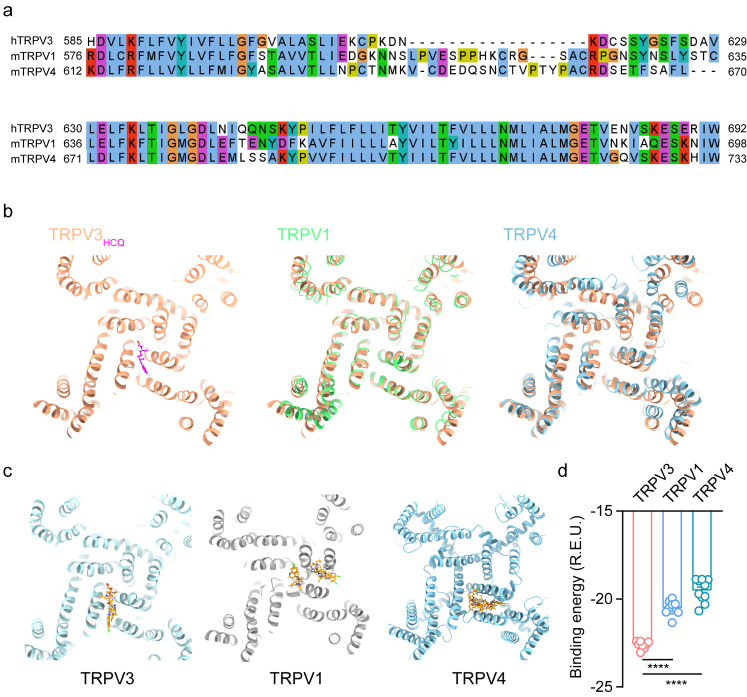


**Fig. S2:**
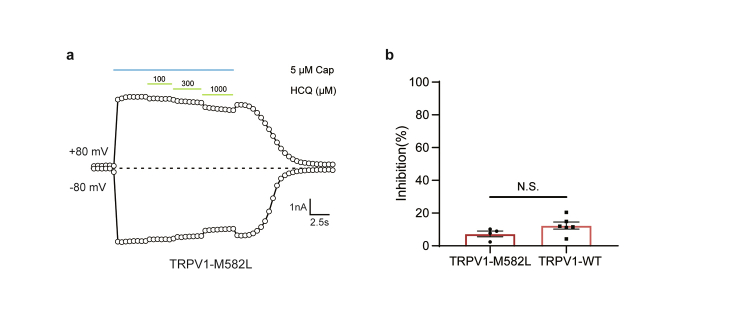


**Fig. S3:**