# Supplementary material for: Inhibition of transient receptor potential vanilloid 3 channels by antimalarial hydroxychloroquine alleviates TRPV3-dependent dermatitis
Source: J Biol Chem. 2024 Sep 2;300(10):107733. doi: 10.1016/j.jbc.2024.107733 (PMC11460631; doi:10.1016/j.jbc.2024.107733)
Supplement: Supplementary Table 1 [file mmc1.docx]

**Supplementary Table1 Roles of TRP channels in atopic dermatitis.**

| Channel name | Expression | Function in AD | Relative cytokines | Reference |
| --- | --- | --- | --- | --- |
| TRPV3 | Keratinocyte, neurons | epidermal hyperkeratosis, itch, inflammation | IL-6, IL-31, TNF-α | Qu Y et al. (1) ；Larkin C et al. (2) ；Qu Y et al. (3) ； |
| TRPA1 | neurons | Itch, neurogenic inflammation | TSLP, IL-4, IL-13, IL-31 | Wilson SR et al. (4) ；Oetjen LK et al. (5) ；Oh MH et al. (6) ；Cevikbas F et al. (7) ； |
| TRPV1 | Neurons, keratinocyte | Itch, neurogenic inflammation, pain | IL-4, IL-13, IL-31, TNF-α | Oetjen LK et al. (5) ； Cevikbas F et al. (7) ；Khan AA et al. (8) |
| TRPV4 | Neurons, keratinocyte, mast cell | Allergies, itch | IL-17a | Zhang Q et al. (9) ；Segond von Banchet G et al. (10) |
| TRPM8 | neurons， melanocyte | relieve itch | serotonin | Sanders KM et al. (11) |

**Reference**

1. Qu, Y., Sun, X., Wei, N., and Wang, K. (2023) Inhibition of cutaneous heat-sensitive Ca(2+) -permeable transient receptor potential vanilloid 3 channels alleviates UVB-induced skin lesions in mice. *FASEB journal : official publication of the Federation of American Societies for Experimental Biology* **37**, e23309

2. Larkin, C., Chen, W., Szabó, I. L., Shan, C., Dajnoki, Z., Szegedi, A., Buhl, T., Fan, Y., O'Neill, S., Walls, D., Cheng, W., Xiao, S., Wang, J., and Meng, J. (2021) Novel insights into the TRPV3-mediated itch in atopic dermatitis. *The Journal of allergy and clinical immunology* **147**, 1110-1114.e1115

3. Qu, Y., Wang, G., Sun, X., and Wang, K. (2019) Inhibition of the Warm Temperature-Activated Ca(2+)-Permeable Transient Receptor Potential Vanilloid TRPV3 Channel Attenuates Atopic Dermatitis. *Molecular pharmacology* **96**, 393-400

4. Wilson, S. R., Thé, L., Batia, L. M., Beattie, K., Katibah, G. E., McClain, S. P., Pellegrino, M., Estandian, D. M., and Bautista, D. M. (2013) The epithelial cell-derived atopic dermatitis cytokine TSLP activates neurons to induce itch. *Cell* **155**, 285-295

5. Oetjen, L. K., Mack, M. R., Feng, J., Whelan, T. M., Niu, H., Guo, C. J., Chen, S., Trier, A. M., Xu, A. Z., Tripathi, S. V., Luo, J., Gao, X., Yang, L., Hamilton, S. L., Wang, P. L., Brestoff, J. R., Council, M. L., Brasington, R., Schaffer, A., Brombacher, F., Hsieh, C. S., Gereau, R. W. t., Miller, M. J., Chen, Z. F., Hu, H., Davidson, S., Liu, Q., and Kim, B. S. (2017) Sensory Neurons Co-opt Classical Immune Signaling Pathways to Mediate Chronic Itch. *Cell* **171**, 217-228.e213

6. Oh, M. H., Oh, S. Y., Lu, J., Lou, H., Myers, A. C., Zhu, Z., and Zheng, T. (2013) TRPA1-dependent pruritus in IL-13-induced chronic atopic dermatitis. *Journal of immunology (Baltimore, Md. : 1950)* **191**, 5371-5382

7. Cevikbas, F., Wang, X., Akiyama, T., Kempkes, C., Savinko, T., Antal, A., Kukova, G., Buhl, T., Ikoma, A., Buddenkotte, J., Soumelis, V., Feld, M., Alenius, H., Dillon, S. R., Carstens, E., Homey, B., Basbaum, A., and Steinhoff, M. (2014) A sensory neuron-expressed IL-31 receptor mediates T helper cell-dependent itch: Involvement of TRPV1 and TRPA1. *The Journal of allergy and clinical immunology* **133**, 448-460

8. Khan, A. A., Diogenes, A., Jeske, N. A., Henry, M. A., Akopian, A., and Hargreaves, K. M. (2008) Tumor necrosis factor alpha enhances the sensitivity of rat trigeminal neurons to capsaicin. *Neuroscience* **155**, 503-509

9. Zhang, Q., Henry, G., and Chen, Y. (2021) Emerging Role of Transient Receptor Potential Vanilloid 4 (TRPV4) Ion Channel in Acute and Chronic Itch. *International journal of molecular sciences* **22**

10. Segond von Banchet, G., Boettger, M. K., König, C., Iwakura, Y., Bräuer, R., and Schaible, H. G. (2013) Neuronal IL-17 receptor upregulates TRPV4 but not TRPV1 receptors in DRG neurons and mediates mechanical but not thermal hyperalgesia. *Molecular and cellular neurosciences* **52**, 152-160

11. Sanders, K. M., Hashimoto, T., Sakai, K., and Akiyama, T. (2018) Modulation of Itch by Localized Skin Warming and Cooling. *Acta dermato-venereologica* **98**, 855-861
