# Supplementary material for: Inhibition of transient receptor potential vanilloid 3 channels by antimalarial hydroxychloroquine alleviates TRPV3-dependent dermatitis
Source: J Biol Chem. 2024 Sep 2;300(10):107733. doi: 10.1016/j.jbc.2024.107733 (PMC11460631; doi:10.1016/j.jbc.2024.107733)
Supplement: Supporting Information [file mmc2.docx]

**Fig S1. Molecular docking of HCQ to TRPV3, TRPV1 and TRPV4.** (a) Sequence alignment of TRPV3, TRPV1, and TRPV4. (b) Structure differences of HCQ binding pockets on TRPV3, TRPV1, and TRPV4. (c) Top 10 docking models with the best binding energy of HCQ on TRPV3, TRPV1, and TRPV4. (d) Binding energies of top 10 docking models from panel **c**.

**Fig. S2 The effect of HCQ on TRPV1 M582L mutant.**

**Fig. S3 Wash-off speed of 1 mM 2-APB and 1 mM carvacrol.** (a) wash-off of TRPV3 current induced by 1 mM 2-APB or 1 mM carvacrol. (b) Quantification of wash-off time constant tau. Data were represented as mean ± s. e. m. (n = 5 cells). Data points of every cell were conducted by calculating the average of three repeated perfusions from panel **a**. **, p =0.0010 by paired two-tailed t-test.

**Table S1 Roles of TRP channels in atopic dermatitis.**
